# Preoperative kidney tumor risk estimation with AI: From logistic regression to transformer

**DOI:** 10.1371/journal.pone.0323240

**Published:** 2025-05-30

**Authors:** Vesna Barros, Nour Abdallah, Michal Ozery-Flato, Avihu Dekel, Moshiko Raboh, Nicholas Heller, Simona Rabinovici-Cohen, Alex Golts, Amilcare Gentili, Daniel Lang, Suman Chaudhary, Varsha Satish, Resha Tejpaul, Ivan Eggel, Itai Guez, Ella Barkan, Henning Müller, Efrat Hexter, Michal Rosen-Zvi, Christopher Weight

**Affiliations:** 1 IBM Research Israel, Haifa, Israel; 2 The Hebrew University of Jerusalem, Jerusalem, Israel; 3 Glickman Urological and Kidney Institute, Cleveland Clinic, Cleveland, Ohio, United States of America; 4 University of Minnesota, Minneapolis, Minnesota, United States of America; 5 San Diego VA Health Care System, San Diego, California, United States of America; 6 Institute of Radiation Medicine, Helmholtz Munich, Munich, Germany; 7 Physics Department, Technical University of Munich, Garching, Germany; 8 Taiyuan University of Technology, Taiyuan, China; 9 Indian Institute of Technology Bombay, Bombay, India; 10 University of Applied Sciences Western Switzerland (HES-SO), Sierre, Switzerland; 11 University of Geneva, Geneva, Switzerland; Katholieke Universiteit Leuven UZ Leuven: Katholieke Universiteit Leuven Universitaire Ziekenhuizen Leuven, BELGIUM

## Abstract

We consider the problem of renal mass risk classification to support doctors in adjuvant treatment decisions following nephrectomy. Recommendation of adjuvant therapy based on the mass appearance poses two major challenges: first, morphologic patterns may sometimes overlap across subtypes of varying risks. Second, interobserver variability is large. These complexities encourage the use of computational models as accurate noninvasive tools to find relevant relationships between individual perioperative renal mass characteristics and patient risk. In addition, recent evidence highlights the importance of clinical context as a promising direction to inform treatment decisions post-nephrectomy. In this work, we aim to identify relevant clinical markers that can be predictive of renal cancer prognosis. As a starting point, we perform a clinical feature ablation study by training a logistic regression baseline model to predict renal cancer patients’ eligibility for adjuvant therapy. The training dataset consisted of medical records of 300 individuals with renal tumors who underwent partial or radical nephrectomy between 2011 and 2020. In addition, we evaluate the same task using a transformer-based model pretrained on a much larger dataset of over 300,000 clinical records of individuals from the UK Biobank. Our findings demonstrate the pretrained model’s efficacy in knowledge transfer across different populations, with radiographic data from preoperative cross-sectional imaging playing an important role in informing renal risk and treatment decisions.

## Introduction

Renal tumor treatment decisions depend on evaluating the pathologic attributes of the tumor and perioperative treatment outcomes, yet accurately classifying tumor mass risk remains challenging due to variability in morphologic patterns and the substantial interobserver variability among medical urologic pathologists [1,2]. The advancement of artificial intelligence (AI) holds promise for automating these evaluations and acquiring the ability to extract features that might be subtle or even imperceptible to human readers. Presently, AI methods primarily focus on renal cancer diagnosis, employing deep learning models as second readers to aid radiologists in image interpretation [3–5]. However, most models consider only pixel-value information without informing clinical context. In practice, relevant and precise non-imaging data derived from clinical history and laboratory findings empower physicians to contextualize imaging results effectively, leading to improved diagnostic accuracy and more informed clinical decision-making [6].

Despite the recent progress in automatic kidney tumor diagnosis, the findings of studies that applied AI to recommend adjuvant treatment directly have not previously been well-established in the literature. Existing work relates to predicting tumor aggressiveness [7] or performing kidney segmentation [8,9] as an alternative method to stage kidney tumors. Both applications aim to quantify the complexity of renal masses radiographically for improved treatment decision-making. However, an objective and standardized tool to reliably aid in patient risk stratification based on information from multiple data modalities is still missing.

To overcome this limitation, the **K**idney Clinical **N**otes and **I**maging to **G**uide and **H**elp Personalize **T**reatment and Biomarkers Discovery (abbreviated KNIGHT) Challenge [10] was proposed in conjunction with the 2022 IEEE International Symposium on Biomedical Imaging (ISBI) [11]. In the Challenge, teams used the publicly available KiTS dataset [12] of computed tomography (CT) imaging of the kidneys and corresponding patient clinical data to develop the best AI models to predict the risk class of patients with renal masses. Developing such tools has important clinical implications for advancing patient care, as high-risk patients often require adjuvant treatment to prevent recurrence and improve overall survival [13]. To facilitate this, the Challenge categorized lower-risk and higher-risk groups and proposed a binary patient classification based on the need for adjuvant therapy. Notably, upon analyzing their performance, the winning team achieved remarkable results using solely clinical data, surpassing others that utilized both clinical and imaging data.

While employing diverse methodologies, with a primary focus on deep learning, none of the KNIGHT Challenge teams explored large pretrained models based on the transformer architecture [14]. Transformers are a type of deep learning architecture designed to process sequential data. It does so by using the self-attention mechanism to weigh the importance of each input element relative to others in a sequence. This allows transformers to capture long-range dependencies and contextual relationships more effectively. Transformer-based architectures have demonstrated remarkable efficacy in handling electronic health records for disease predictions [15–17]. Their effectiveness lies in their ability to transfer knowledge obtained from extensive datasets to smaller target datasets through a pretraining process. The knowledge gained during the pretraining phase is then leveraged to finetune the model for the downstream tasks. In scenarios where data from a few hundred patients are available, such as in the KiTS data, pretraining has the potential to yield substantial performance advantages. Thus, we explored a few-shot learning scenario, where only a small amount of downstream task data is available for finetuning the models. Specifically, few-shot learning focuses on leveraging knowledge gained from other tasks to effectively generalize and perform well on new, downstream predictive tasks.

Building on these insights, this paper aims at (1) summarizing the main outcomes of the KNIGHT Challenge, (2) performing an ablation study with the KiTS clinical data to investigate clinical markers predictive of renal cancer risk and (3) evaluating the effectiveness of a transformer-based model to outperform the winning team and to transfer knowledge from a much larger dataset, the UK Biobank, to the KiTS dataset through its pretraining process. We analyze the performance of the pretrained model in a few-shot learning setting, i.e., when few labeled data are available for finetuning.

We found that although imaging data plays a role in predicting outcomes, using clinical history and visual features extracted from radiological images, such as tumor size from preoperative CT scans, may suffice to achieve comparable outcome predictions. While the pretrained model did not surpass the KNIGHT winning model, mainly due to the absence of pretraining with radiological tumor size, we highlight the broader potential of pretrained models to learn robust representations and transfer knowledge from one dataset to another. Finally, we introduce the AI systems developed in the Challenge as benchmark models, facilitating fair comparisons with future studies that pursue addressing similar tasks using different methods or datasets.

## Materials and methods

This retrospective study was approved by the institutional review board from the University of Minnesota - Twin Cities (protocol code 1611M00821). The KiTS dataset was collected and maintained over several years (S1 Appendix). This research has also been conducted using the UK Biobank Resource under Application Number 95318. Data for the current analysis was downloaded on July 1st, 2021. This study was covered by the generic ethical approval for UK Biobank studies from the National Research Ethics Service Committee North West–Haydock (approval letter dated 29th June 2021, Ref 21/NW/0157), and all study procedures were performed following the World Medical Association Declaration of Helsinki ethical principles for medical research.

The KNIGHT baseline model is available at https://github.com/BiomedSciAI/fuse-med-ml/tree/master/fuse_examples/imaging/classification/knight.

### KiTS data collection

**Fig 1:**
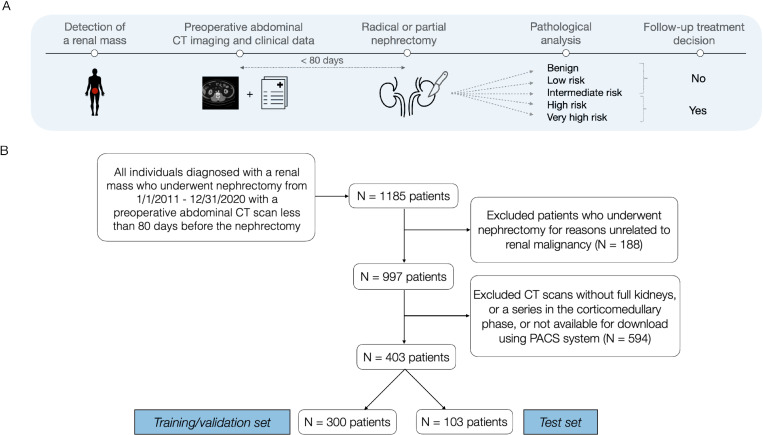
Patient journey and data collection. **(A)** An abdominal CT scan must have happened within 80 days before nephrectomy. Follow-up treatment decisions and risk categorization were derived from postoperative pathology results. **(B)** Flowchart with selection criteria for the study sample of 403 individuals at either Fairview University of Minnesota Medical Center or Cleveland Clinic in Ohio, USA. PACS = Picture Archiving and Communication System.

**Fig 2:**
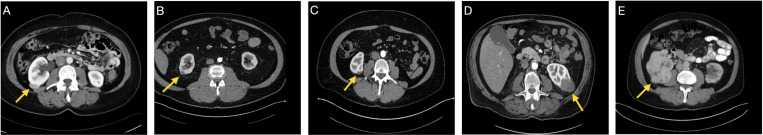
Example of CT images for each risk category with information regarding the finding size. Radiographic findings (tumors or benign) are indicated with arrows. **(A)** Benign: Image of a 44-year-old woman showing a 2.4 cm angiomyolipoma. **(B)** Low risk: Image of a 50-year-old man showing a 2.2 cm papillary renal cell carcinoma (RCC). **(C)** Intermediate risk: Image of a 49-year-old man showing a 2.4 cm clear cell RCC. **(D)** High risk: Image of a 74-year-old man showing a 4.5 cm papillary RCC. **(E)** Very high risk: Image of a 68-year-old man showing a 10.6 cm clear cell RCC.

The cohort was composed of individuals who underwent partial or radical nephrectomy between 2011 and 2020 to excise a renal tumor at either Fairview University of Minnesota Medical Center or Cleveland Clinic in Ohio, USA (Fig 1A). Data collection was conducted in three distinct phases throughout nine years (S1 Fig). A single combined flowchart with the eligibility criteria is exemplified in Fig 1B. We included 403 patients with preoperative abdominal CT imaging in the late-arterial phase and available clinical information. Patients who underwent nephrectomy for transplant purposes were excluded. Risk classification labels deduced from postoperative pathology results were benign, low-risk, intermediate-risk, high-risk, and very high-risk (Fig 2). To improve statistical power and ensure reliable predictions in the Challenge, these risk classes were grouped into two larger categories based on the follow-up treatment: lower-risk classes such as benign, low-risk, and intermediate-risk are not candidates for adjuvant therapy, whereas the higher-risk classes high-risk and very high-risk are. In the KNIGHT Challenge, participants were given data from 300 patients (mean age, 58 years ± 15), which were divided into training and validation according to their criteria (Table 1). The test set used for evaluation consisted of another 103 patients selected with similar criteria (mean age, 63 years ± 12). S1 Appendix describes the clinical data in detail, including a description of features and missing values (S1 Table).

**Table 1:** Patient characteristics of training and validation (development) and test sets of KiTS dataset.

	Development set (training and validation)	Test set
Number of patients	300	103
Number of women	120 (40.0)	36 (35.0)
Age at nephrectomy (year)^*^	58 ± 15	63 ± 12
Most recent body mass index (kg/m^2^)^*^	30.9 ± 6.7	31.4 ± 8.0
Preoperative eGFR (mL/min/1.73m^2^) ^*^	68.8 ± 13.4	67.3 ± 15.5
Smoking history	163 (54.3)	58 (56.3)
Myocardial infarction history	13 (4.3)	7 (6.8)
Outcome/Risk group		
Adjuvant therapy candidacy	87 (29.0)	28 (27.2)
Benign	25 (8.3)	6 (5.8)
Low risk	134 (44.7)	47 (45.6)
Intermediate risk	54 (18.0)	21 (20.4)
High risk	41 (13.7)	10 (9.7)
Very high risk	46 (15.3)	18 (17.5)

Note: Data in parenthesis represent percentages.

* Data represent mean ± standard deviation.

eGFR = estimated glomerular filtration rate.

### Assessment of KNIGHT Challenge models performance

In the KNIGHT Challenge, several participants trained models using diverse methodologies with a primary focus on deep learning (Table 2) and submitted a continuous score representing the degree to which patients belong to the adjuvant therapy eligibility class. During submission, they could compare their results to a benchmark model developed by IBM Research built upon the FuseMedML [18] framework.

**Table 2:** The methodology proposed by the IBM Research benchmark model and the four best teams that exceeded the benchmark performance.

Team	Used imaging data	Used clinical data	Used imaging segmentation	Model description
1^st^ place: Amilcare Gentili, University of California San Diego	✗	✓	✗	Ensemble of different models with AutoGluon autoML^1^ using a subset of clinical data.
2^nd^ place: Daniel Lang et al., Helmholtz Center Munich	✓	✓	✓	A 2D U-Net was trained for segmentation. Two patches (left and right kidneys) are fed into convolutional networks, deep features are concatenated with subset of clinical features [19].
3^rd^ place: Suman Chaudhary et al., Taiyuan University of Technology	✗	✓	✗	Classification model based on deep tabular data learning architecture (TabNet) using clinical data only [20].
4^th^ place: Varsha Satish et al., Indian Institute of Technology Bombay	✓	✓	✓	An attention-based deep learning framework that fuses both the clinical and CT imaging features [21].
Challenge benchmark model: IBM Research	✓	✓	✗	CT imaging is input to a 3D ResNet-18 and clinical data is fed into a multilayer perceptron (MLP). Both output representations are concatenated and fed into another MLP that learns the outcome [10].

^1^ https://auto.gluon.ai/

In total, four teams outperformed the benchmark. We normalized their scores by rescaling in the range [0,1]. Given that ensemble models could generate more accurate predictions than individual classifiers in previous clinical applications [22], we explored the robustness of an ensemble model based on the average predictions of the benchmark and the four models, aiming to evaluate whether combining multiple predictions could enhance reliability and better reflect real-world clinical scenarios.

### Clinical feature ablation study

We performed an ablation study with the KiTS data to determine the feature sets that predominantly influence kidney tumor treatment recommendations. To this end, we trained a logistic regression for adjuvant therapy prediction based only on the patient’s preoperative clinical features (see details on the training in S2 Appendix). We chose a logistic regression as a baseline for all experiments in this study, as our goal was not to outperform the Challenge’s models but to quantify the contribution of individual features to the prediction. We randomly split the development set into training (80%) and validation (20%) sets and assessed the predictive power of the baseline on the test set. We grouped the KiTS clinical features into five groups (Table 3) and compared the performance of the classifier trained with all features versus individual feature sets. Finally, we analyzed local explanations in the validation set using SHapley Additive exPlanations (SHAP) [23] due to its capacity to provide reliable local explanations and its solid theory foundations derived from axioms of game theory [24].

**Table 3:** Feature sets for clinical ablation study.

Group 1 Demographics	Age at nephrectomy, gender.
Group 2 Social determinants of health	Chewing tobacco use, alcohol level, smoking level, smoking history, alcohol consumption.
Group 3 Comorbidities	Myocardial infarction, congestive heart failure, peripheral vascular disease, cerebrovascular disease, dementia, chronic obstructive pulmonary disease, connective tissue disease, peptic ulcer disease, uncomplicated diabetes mellitus, diabetes mellitus with end organ damage, chronic kidney disease, hemiplegia from stroke, leukemia, malignant lymphoma, localized solid tumor, metastatic solid tumor, mild liver disease, moderate to severe liver disease, AIDS.
Group 4 Clinical measurements	Preoperative estimated glomerular filtration rate (eGFR) value (ml/min), body mass index (kg/m^2^), body mass index category, days before nephrectomy at which eGFR was measured.
Group 5 Visual features	Radiographic size, R.E.N.A.L. nephrometry score^*^.

* The R.E.N.A.L. nephrometry score was provided to us by the KNIGHT competition organizers solely for the purpose of the clinical ablation study and was not used in the competition. The only radiographic feature utilized by the competition teams was the tumor size.

### Transformer-based model

Given that none of the KNIGHT Challenge teams explored transformer-based methods, we investigated whether this architecture would provide comparable outcome prediction performance to that of the winning team. We used BERT [25], a transformer-based deep learning model designed for natural language processing tasks, as the encoder to learn patient representations from the UK Biobank (UKB) population. The UKB is a large-scale prospective study that recruited approximately half a million individuals aged 40–69 years from the general population of the United Kingdom between 2006 and 2010. Detailed information on the study recruitment and clinical information collection can be found in [26]. We used the data from the first visit of the individuals to the assessment centers, which included questionnaires, family history of major diseases, sociodemographic status, early life exposures, physical measures, and results from blood, urine, and saliva assays. We split the UKB cohort into training, validation, and test sets in a ratio of 60:20:20 (S3 Table) and performed hyperparameter optimization to find the best network parameters based on the total pretraining loss in the validation set. The parameters tuned were the learning rate, masking probability, token dimension, neural network depth, number of heads, and the multilayer perceptron dimension. The preprocessing steps used to transform the raw UKB data to the final processed data and a detailed description of the losses can be found in S3 Appendix.

#### Semi-supervised pretraining in the UK Biobank data.

Two essential components of the BERT pretraining process include the masked language model (MLM) and next-sentence prediction. In MLM, a portion of words in a text are randomly replaced with a [MASK] token, and the objective is to predict the original tokens that have been masked. Next sentence prediction involves determining the likelihood of one sentence following another in a given text. In our study, we used MLM to randomly mask 12% of the input tokens of the model and computed the cross-entropy loss over the masked tokens. In addition, we performed a binary classification task to learn the relationship between the clinical features and multiple clinical outcomes. We defined 16 outcomes corresponding to the different clinical conditions in the Charlson Comorbidity Index [27], with a positive outcome being the new diagnosis of the specific condition within a 2-year follow-up period (S4 Table). We employed the macro AUC (area under the receiver operating characteristic curve) as our evaluation metric, aggregating the average of individual AUCs for each clinical condition (S3 Fig).

#### Finetuning for adjuvant treatment prediction in the KiTS dataset.

Following the pretraining process, the model is fine-tuned for adjuvant therapy prediction in the KiTS dataset. To adapt the pretrained model on the UKB data to the adjuvant prediction task on the KiTS dataset, we ensured feature compatibility by identifying the intersection of features between the datasets. All clinical features from the KiTS could be mapped to the same or similar features in the UKB (S5 Table), except the radiographic size and the R.E.N.A.L score, which were not used during pretraining. In addition, the estimated glomerular filtration rate (eGFR) value was estimated for all individuals in the UKB population using the CKD-EPI 2021 creatinine equation [28].

When finetuning, we froze the backbone parameters of the pretrained model and updated only the weights of the last linear layer. Following recent studies [29,30] we assessed the performance of the model in a few-shot learning scenario, varying the finetuning training set from two labeled patients up to the entire training set of 300 patients. To make the classification decision, we used the CLS token output as a global representation of the data that aggregates the information obtained during pretraining.

### Statistical analysis

The estimation of the univariate association between feature distributions and treatment outcome was done with Fisher’s exact test for categorical features and Student’s t-test for continuous features. We corrected multiple hypotheses using the false discovery rate (FDR) correction (P < .05 indicated statistical significance). The logistic regression model’s hyperparameters were tuned with a five-fold cross-validation on the development dataset via a randomized grid search. Sensitivity and specificity were calculated by defining an optimal operating point using Youden’s J statistic [31] in the validation set and then applied to the test set. For univariate analysis and the classifier, we used the Python SciPy library (version 1.7.3; https://scipy.org) and scikit-learn library (version 1.0.2; https://scikit-learn.org), respectively.

To compare the Challenge models’ performances, we used the AUC with 95% DeLong confidence intervals (CI) [32]. In the bootstrap evaluation, CIs were calculated with 1,000 empirical bootstrap replicates of the models’ predictions.

## Results

### Demographic and clinical characteristics of the study patients

Among the 1,185 patients who underwent a nephrectomy between January 2011 and December 2020, we excluded 188 individuals who had a renal mass diagnosis but underwent a nephrectomy for reasons unrelated to renal malignancy (e.g., due to the presence of calculi or retroperitoneal masses found in non-functioning kidneys) (Fig 1B). Next, the remaining 997 patients’ charts were manually reviewed to ensure that CT scans showed the full kidneys and a series in the corticomedullary phase. Finally, 403 qualifying patients were randomly ordered and assigned into development (300 patients) and test sets (103 patients).

To understand the disparities between lower and higher-risk patients, we performed a univariate analysis between candidate and non-candidate patients for adjuvant therapy (Table 4). The size of the tumor reported in the radiologic report (radiographical size) was larger in the group of patients that needed adjuvant therapy (P < 0.001), and the presence of metastatic solid tumors was also more frequent in this group (P < 0.001). The distribution of established risk factors from the literature differed significantly. For instance, older patients were associated with more aggressive tumors than younger ones (mean age 62.7 years vs. 58.6 in the candidate and non-candidates groups, respectively, P = 0.013). Interestingly, body mass index was negatively associated with tumor risk (29.6 kg/m^2^ ± 6.2 vs. 31.6 kg/m^2^ ± 7.3 in candidate and non-candidates groups, respectively, P = 0.02). On the other hand, tobacco smoking and alcohol consumption did not show a significant association with patients’ treatment outcomes.

**Table 4:** The relationship between patient demographics and medical history and adjuvant therapy candidacy (high- and very high-risk patients) in the KNIGHT dataset.

	No. (%) of patients	Adjuvant therapy	No adjuvant therapy	p-value (FDR corrected)
Total population	403	115 [51, 64]^*^	287 [31, 181, 75]^†^	–
Age at nephrectomy (years)	400 (99.3)	62.7 ± 12.7	58.6 ± 13.4	.013
History of myocardial infarction	20 (5.0)	4 (3.5)	16 (5.6)	.635
History of non-renal localized solid tumor	61 (15.1)	17 (14.8)	44 (15.3)	1.000
History of uncomplicated diabetes mellitus	81 (20.1)	25 (21.7)	56 (19.4)	0.802
History of congestive heart failure	16 (4.0)	5 (4.3)	11 (3.8)	0.847
Radiographic size	399 (99.0)	7.6 ± 3.8	3.9 ± 2.2	< 0.001
Preoperative eGFR value (ml/min)	241 (59.8)	67.5 ± 13.1	69.7 ± 14.5	0.372
Body mass index (kg/m^2^)	402 (99.8)	29.6 ± 6.2	31.6 ± 7.3	0.020
Number of women	156 (38.7)	35 (30.4)	121 (42.0)	0.072
Alcohol consumption	232 (57.6)	68 (59.1)	164 (56.9)	0.847
History of chronic obstructive pulmonary disease	15 (3.7)	5 (4.3)	10 (3.5)	0.847
Has smoking history	221 (54.8)	68 (59.1)	153 (53.1)	0.359

Note: Data in parenthesis are percentages. Continuous variables are reported as mean ± standard deviation.

* Numbers in square brackets represent the total number of high-risk and very high-risk patients.

† Numbers in square brackets represent the total number of benign, low-risk, and intermediate-risk patients.

The risk labels sum up to 402 patients as one patient has an unknown risk.

### AI models performance evaluation

**Fig 3:**
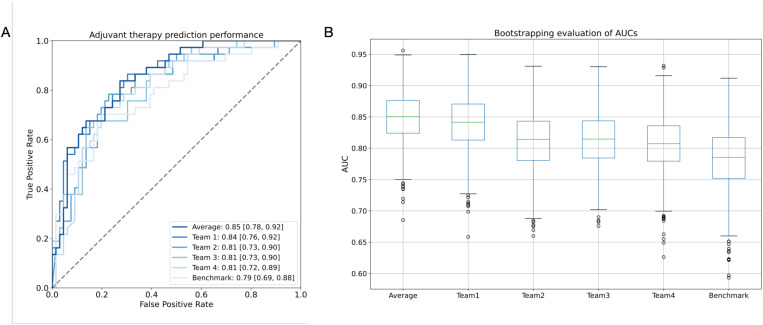
Model performance on the task of adjuvant therapy prediction on the test set. **(A)** Receiver operating characteristic curve (AUC) of the best performing models, average, and benchmark. **(B)** Bootstrapping evaluation of estimated AUCs obtained with 1,000 bootstrap replicates.

The evaluation of the best models’ performance in terms of the AUC with DeLong CI is shown in Fig 3A. The winning team achieved an AUC of 0.84 (95% CI: 0.76, 0.92) in the task of adjuvant therapy prediction, whereas the ensemble average model achieved an AUC of 0.85 (95% CI: 0.78, 0.92). Although we found no evidence of a difference between them (P = 0.847), the bootstrapping evaluation (Fig 3B) indicates that averaging the probabilities resulted in marginally narrower CIs. We analyzed the models’ calibration curves by examining the relationship between the predictions and the ground truth labels on the test set (S4 Fig, S4 Appendix). To quantify this relationship, we computed the Brier score [33] which measures the mean squared difference between the predicted probability and the actual outcome. The average model’s predictions resulted in a lower Brier score compared to other teams, indicating its robustness and well-calibrated probabilities.

### Feature importance analysis

**Fig 4:**
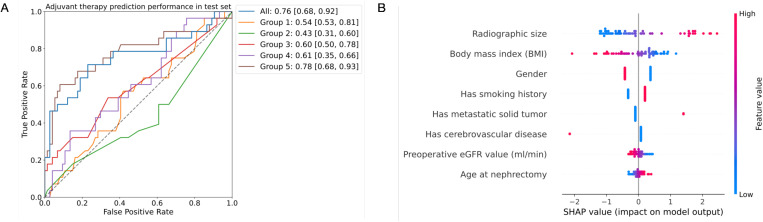
Ablation study results of logistic regression on the adjuvant therapy candidacy prediction task. **(A)** Area under the receiver operating characteristic curves (AUCs) on the test set. Using solely visual features (group 5) produced comparable results to the full set of features (P = 0.671). **(B)** Clinical feature contribution analysis. The features listed in the y-axis are ranked according to their mean absolute impact on the prediction of adjuvant treatment, the top one being the highest contributor to the prediction.

When using all clinical features, the logistic regression achieved an AUC of 0.76 (95% CI: 0.68, 0.92) in the task of adjuvant therapy prediction in the test set (Fig 4A). When using only feature groups 1, 2, 3, 4, or 5, the AUCs were 0.54 (95% CI: 0.53, 0.81), 0.43 (95% CI: 0.31, 0.60), 0.60 (95% CI: 0.50, 0.78), 0.61 (95% CI: 0.35, 0.66) and 0.78 (95% CI: 0.68, 0.93), respectively. The model with all features significantly outperformed all models with individual feature groups 1, 2, 3, and 4 (P = 0.001, P = 0.000, P = 0.028, and P = 0.038, respectively), but the improvement was not significant for the model using only visual features (group 5, P = 0.672). Similar results were encountered for the validation set (S2 Fig).

To measure the ability of logistic regression to discriminate between lower and higher-risk patients, we computed their sensitivity and specificity (S2 Table). On average, the logistic regression trained with all clinical features correctly classified 19 out of 28 patients (67%) who needed adjuvant treatment at the cost of misclassifying 30 out of 74 (41%) non-candidate patients for therapy. When trained with only feature group 5, the model identified 14 out of 37 (51%) eligible patients and 66 out of 74 (89%) non-eligible patients, respectively.

The feature importance analysis showed that the most contributing feature was radiographic size, i.e., the size of the tumor documented in the radiology report (Fig 4B). Patients with large tumor sizes were more likely to need adjuvant therapy after surgical management. Common risk factors (age, gender, body mass index, smoking) are also critical in predicting follow-up treatment: women and non-smokers were less likely to need therapy, whereas older patients and individuals with lower body mass index values tended to have more aggressive tumors. Similarly, patients with lower preoperative estimated glomerular filtration rate values exhibited more aggressive tumor characteristics.

### Pretrained model evaluation

To evaluate the performance of the pretrained model, we compared it to a logistic regression baseline on the raw KiTS data. To allow a fair comparison, we only included the 34 features that could be mapped to the UKB dataset (S5 Table).

**Fig 5:**
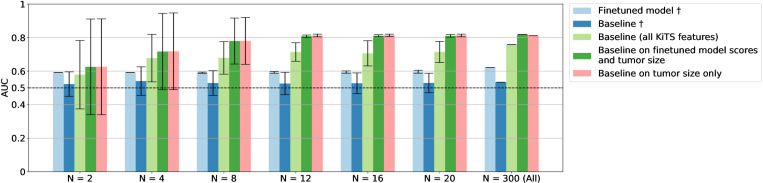
Plots of predictive performance given by the area under the receiver operating curve (AUC) against finetuning training set size on a log scale. Models that did not include tumor size are represented with a ^†^. The finetuned model (light blue bars) outperforms the baseline (dark blue) for all training set sizes, showing that self-supervised pretraining proves to be key in few-shot learning. When adding the tumor size as a feature in the models (light and dark green, and pink bars), performance significantly increases. Ultimately, size alone (pink bar) stood out as the most valuable radiologic feature that can be effectively applied without deep learning models.

Pretraining hyperparameters were optimized to the following final values: max learning rate, 1 × 10–4; learning scheduler, cosine with warmup; optimizer, AdamW with weight decay 0.01; warmup epochs, 5; batch size, 16. Tensorboard was used for experimentation tracking, and the model was pretrained for 60 epochs. We finetuned the pretrained model on randomly selected subsets of increasing size from the KiTS training set. This process results in varying amounts of data available for the models to learn from, going as low as 2 labeled patients up to the entire finetuning training set of 300 patients. Equal class sampling was used to address class imbalance during training, except for the full data where there are only 87 positive training labels. We repeated this process with 20 different random seeds resulting in 20 different AUCs for each training set size. Fig 5 shows the performance curves (average and standard deviation of AUCs) of the experiments. The pretrained model significantly outperformed the baseline on the test set (P < 0.001, paired t-test, light blue versus dark blue bars), achieving an AUC of 0.62 (95% DeLong CI: 0.50, 0.74) when using the full dataset for training, whereas the baseline achieved 0.53 (95% DeLong CI: 0.40, 0.67). While the fine-tuned model demonstrated superior performance, likely due to the patient representation learned during pretraining, its performance remained relatively constant as train data size increased, akin to the behavior of the baseline model.

We then assessed the performance of the baseline model when incorporating all KiTS clinical features, including the patients’ tumor size (light green bars, Fig 5). While this model exhibited a substantial improvement in the average AUC, it also displayed greater variability compared to the baseline model with fewer features. We then trained a logistic regression model on two features: the output score of the finetuned model and the tumor size (dark green bars). This model obtained the largest AUC (0.81 ± 0.007) with only 12 training examples, and this performance remained stable as the training set size increased. Given our findings from the clinical ablation study indicating that radiographical size was the best predictor of tumor risk, we evaluated the baseline solely based on tumor size (as depicted by the pink bars in Fig 5). As expected, tumor size on its own is an excellent predictor, matching the performance of the baseline model using predictions from the pretrained model.

**Fig 6:**
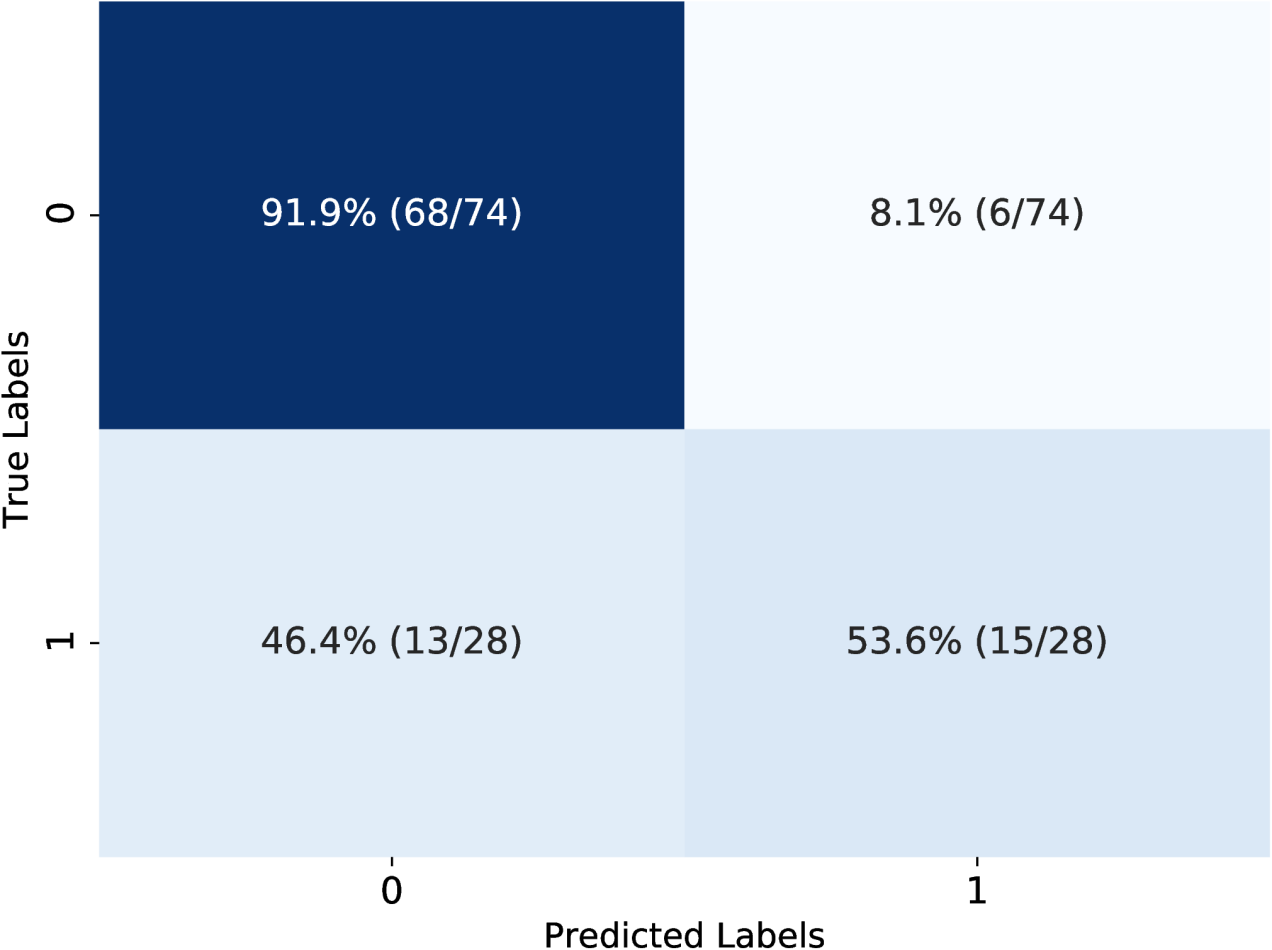
Confusion matrix of the finetuned model representing predicted classification of high-risk cases. Each tile displays the percentage and fraction of patients with lower- and higher-risk renal masses (labels 0 and 1, respectively), stratified by the model’s predictions. The values illustrate the model’s capability to distinguish patients who may require adjuvant therapy. Overall, the model achieved a sensitivity of 53.6%, specificity of 91.9%, and accuracy of 81.4%.

While the AUC is a threshold-independent metric that captures the model’s overall discriminatory ability, we also assessed sensitivity, specificity, and accuracy using a decision threshold of 0.5. These evaluations were conducted on the pretrained model after finetuning with the full training set of 300 patients. The confusion matrix in Fig 6 illustrates the model’s classification performance on the test set. The model correctly identified 91.9% (68/74) of non-aggressive renal cancers that did not require adjuvant therapy, and 53.6% (15/28) of aggressive cases, demonstrating strong specificity and reliable identification of high-risk patients. Additionally, the model achieved a precision of 71.4% (15/21) and an accuracy of 81.4%, highlighting the model’s effectiveness in prioritizing patients who may benefit of adjuvant therapy.

## Discussion

In this study, we investigated clinical markers that contributed to the prediction of renal cancer prognosis. We were motivated by the promising results achieved at the KNIGHT Challenge, where participants developed AI models that recommend treatment planning and performed risk assessments of patients with renal masses. The winning KNIGHT algorithm predicted patients’ adjuvant therapy eligibility with an AUC of 0.84 (95% CI: 0.76, 0.92). Interestingly, it used only patient medical history and the tumor’s size measured in preoperative CT scans, but not imaging data. Although we found no evidence of a difference between the winner and an ensemble average model (AUC = 0.85 [95% CI: 0.78, 0.92], *P* = 0.847), the ensemble model showed marginally better-calibrated probabilities.

In the univariate analysis, we found that older patients tended to belong to higher-risk groups who could benefit from adjuvant treatment. Age is a well-established risk factor for the development of renal cancer [34], but the relationship between age at diagnosis and adjuvant therapy use in the literature is controversial. While some studies state that kidney tumor aggressiveness increases with age, ultimately leading to poor overall survival in older patients [35], other studies show evidence that younger age is associated with unfavorable tumor histological features and an increased number of metastatic sites [36]. Similarly, excessive body weight also represents a strong risk factor for the incidence of renal cell carcinomas [37–39], but multiple studies have demonstrated better prognosis and overall survival in kidney cancer patients with elevated body mass index [40]. The so-called “obesity paradox” refers to the positive link between increased body mass index and improved cancer outcomes. Interestingly, we observed this phenomenon in the clinical feature contribution analysis, where patients with high body mass index values tended not to need additional treatment.

Many few-shot classification algorithms have reported improved performance over the state-of-the-art in several clinical applications [29,30,41]. Their strong performance, however, heavily relies on initially pretraining a network with abundant unlabeled instances with diverse clinical variability. In our study, the pretrained model outperformed the logistic regression baseline on the adjuvant therapy prediction task, even with a limited number of fine-tuning samples. The model’s ability to derive patient representations from the UK Biobank dataset and apply them to predict novel unseen classes in the KiTS dataset shows that it learned useful medical knowledge that can be leveraged to solve downstream population-specific tasks, often characterized by small patient cohorts. After incorporating tumor size in the prediction models, we observed a significant increase in performance, ultimately achieving the largest AUC from all models. This reaffirms that tumor size stands as a valuable radiologic feature that can be effectively applied without the use of deep learning models. It is known that tumor size is a well-established and grounded predictor of several postoperative outcomes within kidney cancer management [42]. However, even when using tumor size as a standalone feature, the learning curve reaches a plateau in performance already at 12 training samples, suggesting that the models might not achieve performance improvement with larger training sets. These findings motivate the collection of other medical modalities, such as molecular data generated by next-generation sequencing techniques or pathological imaging, to enhance diagnostic and prognostic capabilities in renal cancer and link molecular differences between patients to different clinical outcomes. Integrating multimodal modalities is a promising direction for characterizing tumor heterogeneity and informing effective treatment decisions within oncology [43].

Our study had limitations. The test set used for evaluation was relatively small, containing 103 patients. While the dataset is reflective of a diverse patient population commonly encountered in clinical practice, the limited sample size in certain risk groups can affect the generalizability of the results. Social determinants of health such as tobacco and alcohol level consumption were self-reported and thus could be noisy variables. As a retrospective cohort from two institutions, it remains uncertain how effectively the models described here would generalize to medical records from other external sites. Model generalizability is often affected by differences in medical equipment and variations in patient populations across institutions. Such differences have been investigated in previous studies [44,45] and exploring their impact in our model’s performance is an important direction for future work.

Given these findings, there exist several future directions worth pursuing. The true utility of our system is in aiding the physician in treatment decisions for renal tumors, and this requires studies that include such a deployment. While we only predicted higher-risk patients that could benefit from adjuvant treatment, it would be interesting to include in the future other postoperative outcomes such as non-clear cell subtype, survival, recurrence, hospitalization, or readmission. In addition, the pretraining data contained clinical information available from a fixed time point representing the first visit of individuals in the UK Biobank to the assessment centers. Another direction would be incorporating clinical records from other visits and exploring the longitudinal nature of electronic health records. Finally, integrating additional molecular markers could further enhance the AI model’s utility in diagnosis and monitoring of renal tumors. Previous studies have demonstrated that cell-free DNA, circulating tumor DNA, as well as molecular signatures from mRNA, miRNA and proteins provide additional prognostic value in identifying key drivers of aggressive kidney cancers [46,47].

## Conclusion

In summary, the KNIGHT Challenge results support that AI can guide the clinical treatment of kidney tumor patients and aid in discovering new markers. While radiological work primarily focuses on distinguishing malignant from benign tumors, the competition focused on predicting the need for adjuvant treatment after nephrectomy. AI’s potential in healthcare has expanded beyond radiologist-assistive roles [48], with applications such as predicting histopathologic results of breast lesions [44] and breast cancer recurrence after neoadjuvant chemotherapy [49]. Recent studies on AI’s novel tasks highlight new opportunities in medicine, particularly through the success of large pre-trained models (foundation models) in clinical applications. Looking ahead, we propose exploring the integration of these AI technologies and multi-modal healthcare data to enhance predictions of renal cancer outcomes and advance clinical cancer research.

## Supporting information

S1 File S1 Fig. The date span of the data used for the KNIGHT Challenge. S2 Fig. Ablation study results of adjuvant therapy candidacy prediction on the validation set. S3 Fig. Future diagnosis prediction performance in the validation set as a function of training epochs. S4 Fig. Calibration curves on the test set. The average model showed the smallest Brier score. S1 Table. Percentage of missing values in the dataset. S2 Table. Sensitivity and specificity of logistic regression in the test set at Youden’s J-Score operating point selected on the validation set. S3 Table. Data split of the pretraining cohort. S4 Table. Mapping of CCSR codes to 16 Charlson clinical conditions. S1 Appendix. The KiTS database. S2 Appendix. Clinical feature ablation study. S3 Appendix. Pretraining of BERT-based model for clinical records. S4 Appendix. Evaluation of top winners of KNIGHT Challenge.
(ZIP)

## References

[1] Ward RD, Tanaka H, Campbell SC, Remer EM. 2017 AUA renal mass and localized renal cancer guidelines: imaging implications. Radiographics. 2018;38(7):2021–33. doi: 10.1148/rg.2018180127 30339517

[2] Campbell S, Uzzo RG, Allaf ME, Bass EB, Cadeddu JA, Chang A, et al. Renal mass and localized renal cancer: AUA guideline. J Urol. 2017;198(3):520–9. doi: 10.1016/j.juro.2017.04.100 28479239

[3] Deep learning for end-to-end kidney cancer diagnosis on multi-phase abdominal computed tomography. npj Precision Oncol [Internet]. [cited 2024 Jun 2]. Available from: https://www.nature.com/articles/s41698-021-00195-y 10.1038/s41698-021-00195-y PMC8213852 34145374

[4] Zhou L, Zhang Z, Chen Y-C, Zhao Z-Y, Yin X-D, Jiang H-B. A deep learning-based radiomics model for differentiating benign and malignant renal tumors. Transl Oncol. 2019;12(2):292–300. doi: 10.1016/j.tranon.2018.10.012 30448734 PMC6299150

[5] Mahmud S, Abbas TO, Mushtak A, Prithula J, Chowdhury MEH. Kidney cancer diagnosis and surgery selection by machine learning from CT scans combined with clinical metadata. Cancers (Basel). 2023;15(12):3189. doi: 10.3390/cancers15123189 37370799 PMC10296307

[6] Huang SC, Pareek A, Seyyedi S, Banerjee I, Lungren MP. Fusion of medical imaging and electronic health records using deep learning: a systematic review and implementation guidelines. Npj Digit Med. 2020;3(1):1–9. 33083571 10.1038/s41746-020-00341-z PMC7567861

[7] Bektas C, Kocak B, Yardimci A, Turkcanoglu M, Yucetas U, Koca S. Clear cell renal cell carcinoma: machine learning-based quantitative computed tomography texture analysis for prediction of fuhrman nuclear grade. Eur Radiol. 2019;29(3):1153–63. 30167812 10.1007/s00330-018-5698-2

[8] Heller N, Isensee F, Maier-Hein K, Hou X, Xie C, Li F. The state of the art in kidney and kidney tumor segmentation in contrast-enhanced CT imaging: results of the kits19 challenge. Med Image Anal. 2021;67:101821. 33049579 10.1016/j.media.2020.101821 PMC7734203

[9] Golts A, Khapun D, Shats D, Shoshan Y, Gilboa-Solomon F. An ensemble of 3D U-Net based models for segmentation of kidney and masses in CT scans. In: Heller N, Isensee F, Trofimova D, Tejpaul R, Papanikolopoulos N, Weight C, editors. Kidney and kidney tumor segmentation. Cham: Springer International Publishing; 2022. p. 103–15.

[10] IBM Research Haifa. KNIGHT Challenge [Internet]. Available from: https://research.ibm.com/haifa/Workshops/KNIGHT

[11] IEEE International Symposium on Biomedical Imaging 2022 [Internet]. Available from: https://biomedicalimaging.org/2022 10.1109/MPUL.2017.2746243 28961098

[12] Heller N, Sathianathen N, Kalapara A, Walczak E, Moore K, Kaluzniak H, et al. The KiTS19 Challenge data: 300 kidney tumor cases with clinical context, CT semantic segmentations, and surgical outcomes [Internet]. arXiv; 2020 [cited 2024 Jun 2]. Available from: http://arxiv.org/abs/1904.00445

[13] Gul A, Rini BI. Adjuvant therapy in renal cell carcinoma. Cancer. 2019;125(17):2935–44. doi: 10.1002/cncr.32144 31225907

[14] Vaswani A, Shazeer N, Parmar N, Uszkoreit J, Jones L, Gomez AN, et al. Attention is all you need. In: Advances in neural information processing systems [Internet]. Curran Associates, Inc.; 2017 [cited 2024 Jun 2]. Available from: https://proceedings.neurips.cc/paper_files/paper/2017/hash/3f5ee243547dee91fbd053c1c4a845aa-Abstract.html

[15] Li Y, Rao S, Solares J, Hassaine A, Ramakrishnan R, Canoy D. Behrt: transformer for electronic health records. Sci Rep. 2020;10(1):7155. 32346050 10.1038/s41598-020-62922-y PMC7189231

[16] Pang C, Jiang X, Kalluri KS, Spotnitz M, Chen R, Perotte A, et al. CEHR-BERT: incorporating temporal information from structured EHR data to improve prediction tasks [Internet]. arXiv; 2021 [cited 2024 Jun 2]. Available from: http://arxiv.org/abs/2111.08585

[17] Rasmy L, Xiang Y, Xie Z, Tao C, Zhi D. Med-bert: pretrained contextualized embeddings on large-scale structured electronic health records for disease prediction. NPJ Digit Med. 2021;4(1):1–13. 34017034 10.1038/s41746-021-00455-y PMC8137882

[18] Golts A, Raboh M, Shoshan Y, Polaczek S, Rabinovici-Cohen S, Hexter E. FuseMedML: a framework for accelerated discovery in machine learning based biomedicine. JOSS. 2023;8(81):4943. doi: 10.21105/joss.04943

[19] Lang DM, Peeken JC, Combs SE, Wilkens JJ, Bartzsch S. Risk score classification of renal masses on CT imaging data using a convolutional neural network. In: 2022 IEEE International Symposium on Biomedical Imaging Challenges (ISBIC) [Internet]; 2022 [cited 2024 Jun 2]. p. 1–4. Available from: https://ieeexplore.ieee.org/abstract/document/9854698

[20] Chaudhary S, Yang W, Qiang Y. Deep learning-based methods for directing the management of renal cancer using CT scan and clinical information. In: 2022 IEEE International Symposium on Biomedical Imaging Challenges (ISBIC) [Internet]; 2022 [cited 2024 Jun 2]. p. 1–4. Available from: https://ieeexplore.ieee.org/abstract/document/9854722

[21] S V, Nasser SA, Bala G, Kurian NC, Sethi A. Multi-modal information fusion for classification of kidney abnormalities. In: 2022 IEEE International Symposium on Biomedical Imaging Challenges (ISBIC) [Internet]; 2022 [cited 2024 Jun 2]. p. 1–4. Available from: https://ieeexplore.ieee.org/abstract/document/9854644

[22] Altmann A, Rosen-Zvi M, Prosperi M, Aharoni E, Neuvirth H, Schülter E, et al. Comparison of classifier fusion methods for predicting response to anti HIV-1 therapy. PLoS One. 2008;3(10):e3470. doi: 10.1371/journal.pone.0003470 18941628 PMC2565127

[23] Lundberg SM, Lee SI. A unified approach to interpreting model predictions. In: Advances in neural information processing systems [Internet]. Curran Associates, Inc.; 2017 [cited 2024 Jun 2]. Available from: https://proceedings.neurips.cc/paper_files/paper/2017/hash/8a20a8621978632d76c43dfd28b67767-Abstract.html

[24] Malinverno L, Barros V, Ghisoni F, Visonà G, Kern R, Nickel PJ, et al. A historical perspective of biomedical explainable AI research. Patterns (N Y). 2023;4(9):100830. doi: 10.1016/j.patter.2023.100830 37720333 PMC10500028

[25] Devlin J, Chang MW, Lee K, Toutanova K. BERT: pre-training of deep bidirectional transformers for language understanding [Internet]. arXiv; 2019 [cited 2024 Jun 2]. Available from: http://arxiv.org/abs/1810.04805

[26] Sudlow C, Gallacher J, Allen N, Beral V, Burton P, Danesh J, et al. UK biobank: an open access resource for identifying the causes of a wide range of complex diseases of middle and old age. PLoS Med. 2015;12(3):e1001779. doi: 10.1371/journal.pmed.1001779 25826379 PMC4380465

[27] Charlson ME, Pompei P, Ales KL, MacKenzie CR. A new method of classifying prognostic comorbidity in longitudinal studies: development and validation. J Chronic Dis. 1987;40(5):373–83. 3558716 10.1016/0021-9681(87)90171-8

[28] Inker L, Eneanya N, Coresh J, Tighiouart H, Wang D, Sang Y. New creatinine- and cystatin c–based equations to estimate gfr without race. N Engl J Med. 2021;385(19):1737–49. 34554658 10.1056/NEJMoa2102953 PMC8822996

[29] Poulain R, Gupta M, Beheshti R. Few-shot learning with semi-supervised transformers for electronic health records. PMC10399128 37538125

[30] Wornow M, Thapa R, Steinberg E, Fries JA, Shah NH. EHRSHOT: an EHR Benchmark for few-shot evaluation of foundation models [Internet]. arXiv; 2023 [cited 2024 Jun 2]. Available from: http://arxiv.org/abs/2307.0202831

[31] Fluss R, Faraggi D, Reiser B. Estimation of the Youden Index and its associated cutoff point. Biom J. 2005;47(4):458–72. doi: 10.1002/bimj.200410135 16161804

[32] DeLong ER, DeLong DM, Clarke-Pearson DL. Comparing the areas under two or more correlated receiver operating characteristic curves: a nonparametric approach. Biometrics. 1988;44(3):837–45. doi: 10.2307/2531595 3203132

[33] Rufibach K. Use of brier score to assess binary predictions. J Clin Epidemiol. 2010;63(8):938–9. 20189763 10.1016/j.jclinepi.2009.11.009

[34] Lotan Y, Karam J, Shariat S, Gupta A, Roupret M, Bensalah K, et al. Renal-cell carcinoma risk estimates based on participants in the prostate, lung, colorectal, and ovarian cancer screening trial and national lung screening trial. Urol Oncol. 2016;34(4):167.e9–e16. 10.1016/j.urolonc.2015.10.011 26602092

[35] Jiang T, Wu Y-P, Chen S-H, Ke Z-B, Liang Y-C, Xu N. Prognosis and clinicopathological characteristics of renal cell carcinoma: Does bilateral occurrence influence overall and cancer-specific survival? Transl Cancer Res. 2020;9(2):432–40. doi: 10.21037/tcr.2019.11.22 35117388 PMC8798000

[36] Bianchi M, Sun M, Jeldres C, Shariat S, Trinh Q, Briganti A. Distribution of metastatic sites in renal cell carcinoma: a population-based analysis. Ann Oncol. 2012;23(4):973–80. 21890909 10.1093/annonc/mdr362

[37] Shi X, Jiang A, Qiu Z, Lin A, Liu Z, Zhu L, et al. Novel perspectives on the link between obesity and cancer risk: from mechanisms to clinical implications. Front Med. 2024:1–24. 10.1007/s11684-024-1094-2 39542988

[38] Shi X, Deng G, Wen H, Lin A, Wang H, Zhu L, et al. Role of body mass index and weight change in the risk of cancer: a systematic review and meta-analysis of 66 cohort studies. J Glob Health. 2024;14:04067. 38547495 10.7189/jogh.14.04067 PMC10978059

[39] Wen H, Deng G, Shi X, Liu Z, Lin A, Cheng Q, et al. Body mass index, weight change, and cancer prognosis: a meta-analysis and systematic review of 73 cohort studies. ESMO Open. 2024;9(3):102241. doi: 10.1016/j.esmoop.2024.102241 38442453 PMC10925937

[40] Kim LH, Doan P, He Y, Lau HM, Pleass H, Patel MI. A systematic review and meta-analysis of the significance of body mass index on kidney cancer outcomes. J Urol. 2021;205(2):346–55. doi: 10.1097/JU.0000000000001377 32945696

[41] Agrawal M, Hegselmann S, Lang H, Kim Y, Sontag D. Large language models are few-shot clinical information extractors [Internet]. arXiv; 2022 [cited 2024 Jun 2]. Available from: http://arxiv.org/abs/2205.12689

[42] Frank I, Blute ML, Cheville JC, Lohse CM, Weaver AL, Zincke H. Solid renal tumors: an analysis of pathological features related to tumor size. J Urol. 2003;170(6 Pt 1):2217–20. doi: 10.1097/01.ju.0000095475.12515.5e 14634382

[43] Kashyap A, Rapsomaniki MA, Barros V, Fomitcheva-Khartchenko A, Martinelli AL, Rodriguez AF, et al. Quantification of tumor heterogeneity: from data acquisition to metric generation. Trends Biotechnol. 2022;40(6):647–76. doi: 10.1016/j.tibtech.2021.11.006 34972597

[44] Barros V, Tlusty T, Barkan E, Hexter E, Gruen D, Guindy M, et al. Virtual biopsy by using artificial intelligence-based multimodal modeling of binational mammography data. Radiology. 2023;306(3):e220027. doi: 10.1148/radiol.220027 36283109

[45] McKinney SM, Sieniek M, Godbole V, Godwin J, Antropova N, Ashrafian H, et al. International evaluation of an AI system for breast cancer screening. Nature. 2020;577(7788):89–94. doi: 10.1038/s41586-019-1799-6 31894144

[46] Cancer Genome Atlas Research Network Analysis working group, Creighton C, Morgan M, Gunaratne P, Wheeler D, Gibbs R, et al. Comprehensive molecular characterization of clear cell renal cell carcinoma. Nature. 2013;499(7456):43–9. 23792563 10.1038/nature12222 PMC3771322

[47] Green EA, Li R, Albiges L, Choueiri TK, Freedman M, Pal S, et al. Clinical utility of cell-free and circulating tumor DNA in kidney and bladder cancer: a critical review of current literature. Eur Urol Oncol. 2021;4(6):893–903. doi: 10.1016/j.euo.2021.04.005 33975782

[48] Chorev M, Shoshan Y, Akselrod-Ballin A, Spiro A, Naor S, Hazan A. The case of missed cancers: applying AI as a radiologist’s safety net. In: Martel A, Abolmaesumi P, Stoyanov D, Mateus D, Zuluaga M, Zhou S, editors. Medical image computing and computer assisted intervention – MICCAI 2020. Cham: Springer International Publishing; 2020. p. 220–9.

[49] Rabinovici-Cohen S, Fernández XM, Grandal Rejo B, Hexter E, Hijano Cubelos O, Pajula J, et al. Multimodal prediction of five-year breast cancer recurrence in women who receive neoadjuvant chemotherapy. Cancers (Basel). 2022;14(16):3848. doi: 10.3390/cancers14163848 36010844 PMC9405765

