# Supplementary material for: Preoperative kidney tumor risk estimation with AI: From logistic regression to transformer
Source: PLoS One. 2025 May 30;20(5):e0323240. doi: 10.1371/journal.pone.0323240 (PMC12124753; doi:10.1371/journal.pone.0323240)
Supplement: S1 File — S1 Fig. The date span of the data used for the KNIGHT Challenge. S2 Fig. Ablation study results of adjuvant therapy candidacy prediction on the validation set. S3 Fig. Future diagnosis prediction performance in the validation set as a function of training epochs. S4 Fig. Calibration curves on the test set. The average model showed the smallest Brier score. S1 Table. Percentage of missing values in the dataset. S2 Table. Sensitivity and specificity of logistic regression in the test set at Youden’s J-Score operating point selected on the validation set. S3 Table. Data split of the pretraining cohort. S4 Table. Mapping of CCSR codes to 16 Charlson clinical conditions. S1 Appendix. The KiTS database. S2 Appendix. Clinical feature ablation study. S3 Appendix. Pretraining of BERT-based model for clinical records. S4 Appendix. Evaluation of top winners of KNIGHT Challenge. (ZIP) [file pone.0323240.s001.zip › supp.docx]

# **Supporting information**

## **S1 Appendix: The KiTS database**

### **Data collection**

The data used for the KNIGHT Challenge was collected in three distinct phases. The same eligibility criteria were used in all of them, except for the later bound of the period when the data was collected. In each phase, we extended the period to include all patients whose data became available in the time that had passed since the previous phase had closed.

In the data collection process, a query was made against the electronic medical record systems of Fairview University of Minnesota Medical Center and Cleveland Clinic to identify patients who (a) had undergone a partial or radical nephrectomy procedure, and (b) had a CT scan which included the abdomen sometime within the 80 days before their nephrectomy. This query automatically excluded patients who had chosen to opt out of inclusion in retrospective research studies. Next, these patients’ charts were manually reviewed to include only the patients who underwent nephrectomy for a renal mass that was suspected of malignancy. This step excluded patients who had a renal mass diagnosis code but underwent nephrectomy for reasons unrelated to renal malignancy. For instance, many of the masses were adrenal, urothelial, or other retroperitoneal masses that affected the kidney but did not originate from it. Others still underwent nephrectomy because of calculi or other issues that were found in non-functioning kidneys. Finally, the remaining cases were reviewed to ensure their CT scan (a) included all kidneys in full, (b) included a series in the corticomedullary phase, and (c) was available for download using our PACS system.

The first phase took place during the second half of 2018 and its purpose was to collect 210 cases for the training set of the 2019 Kidney Tumor Segmentation Challenge (KiTS19) [1]. In this phase, our query searched for all patients who underwent nephrectomy between Jan. 1, 2011, and May 15, 2018. It returned 798 patients, of whom 657 were deemed to have undergone nephrectomy for fear of renal malignancy. The imaging from 399 cases was further reviewed until 210 were found that included the corticomedullary phase and could be downloaded.

The second phase took place during the summer of 2019 and its purpose was to collect 90 cases for the test set of the KiTS19. In this phase, our query searched for all patients who underwent nephrectomy between Jan. 1, 2011, and June 15, 2018. It returned 962 patients, of whom 799 were deemed to have undergone nephrectomy for fear of renal malignancy. The imaging from 145 cases was further reviewed until 90 were found that included the corticomedullary phase and could be downloaded.

Lastly, the third and final phase took place late in 2021 and its purpose was to collect 103 cases for the test set of the KNIGHT challenge. In this phase, our query searched for all patients who underwent nephrectomy between Jan. 1, 2011, and Dec 31, 2020. It returned 1186 patients, of whom 997 were deemed to have undergone nephrectomy for fear of renal malignancy. The imaging from 168 cases was further reviewed until 103 were found that included the corticomedullary phase and could be downloaded.

To prevent duplication, patients were not considered if they had been selected for one of the prior phases. This caused each phase to overrepresent more recent nephrectomies in each subsequent cohort. This can be seen clearly in S1 Fig.

**S1 Fig.** The date span of the data used for the KNIGHT Challenge.

### **Clinical data description**

Medical records included demographics, risk factors, comorbidities, and CT image-extracted features (see [1] for a complete description of each attribute). In addition, the R.E.N.A.L. nephrometry scores were provided for us by the competition organizers. We used the R.E.N.A.L. score because it was directly extracted from EHR data from Fairview University of Minnesota Medical Center and Cleveland Clinic. We also created a new feature called “body mass index (BMI) category” by thresholding the BMI values into three categories: BMI ≤ 18.5 kg/m^2^ were considered low, 18.5 kg/m^2^ ≤ BMI ≤ 24.9 kg/m^2^ were considered normal, and BMI ≥ 24.9 kg/m^2^ were considered high. Positive surgical margins (PSM), referring to the detection of cancer cells at the edge of the tissue removed during nephrectomy, were present in only 15 out of 300 patients in the training set. Despite the potential impact of PSM on cancer recurrence following partial nephrectomy, certain surgical series have reported minimal or even negligible correlation between PSM and the progression of kidney cancer [2]. Given the low PSM incidence rate in our data and the uncertain association between PSM and outcomes, we decided to not incorporate this information in the data. Finally, we analyzed the proportion of missing data in the entire dataset. The only attributes containing missing values were self-reported features related to alcohol and tobacco consumption, in addition to preoperative estimated glomerular filtration rate (eGFR) and radiographic size. S1 Table shows the percentage of missing values for these features.

**S1 Table. Percentage of missing values in the dataset.**

| Feature | Percentage of missing values |
| --- | --- |
| Chewing tobacco use | 0.5 (2/403) |
| Alcohol level | 42.4 (171/403) |
| Alcohol consumption | 42.4 (171/403) |
| Radiographic size | 1.0 (4/403) |
| Preoperative eGFR value (ml/min) | 40.2 (162/403) |
| Days before nephrectomy at which eGFR was measured | 22.33 (90/403) |
| Smoking level | 0.5 (2/403) |
| Has smoking history | 0.5 (2/403) |
| Risk category | 0.25 (1/403) |

## **S2 Appendix: Clinical feature ablation study**

### **Model training**

During model training, the development dataset of 300 patients was split between train (240 patients) and validation (60 patients) datasets. Imputation for completing missing values was done using k-Nearest Neighbors [3]. Features were transformed by scaling each value to the range (0,1) on the training set and applied to the validation and test sets.

To maximize model performance and prevent overfitting, we tuned the logistic regression hyperparameters with a five-fold cross-validation on the train dataset, via a randomized grid search, and chose the hyperparameters combination that led to the best performance in the validation set. Since our models generated slightly different performances solely based on the random seed used to initialize the optimization procedure, we trained 10 models to account for variability. We reported the performance of a global model obtained with the average of the runs.

We used Python’s library *scikit-learn* to train the logistic regression and to optimize the following parameters:

- Tolerance for stopping criteria (*tol*), varied between 5, 3, 2, 1, 1e-1, 1e-2, and 1e-3.
- Inverse of regularization strength (*C*), varied between 1e-3, 1e-2, 1e-1, 1, 5, 10, 100.

All other parameters were kept as *scikit-learn*’s default values.

### **Model evaluation on the validation set**

The performance curve for each feature group in the validation set can be found in S2 Fig. When using all clinical features, logistic regression achieved an AUC of 0.80 (95% CI: 0.68, 0.92) in the task of adjuvant therapy prediction in the validation set. When using only feature groups 1, 2, 3, 4, or 5, the AUCs were 0.67 (0.53, 0.81), 0.46 (0.31, 0.60), 0.64 (0.50, 0.78), 0.51 (0.35, 0.66) and 0.80 (0.68, 0.93), respectively. The model with all features significantly outperformed all models with individual feature groups 1, 2, 3, and 4 (P = 0.045, P = 0.001, P = 0.036, and P = 0.004, respectively). However, there was no significant performance difference when using only visual features (group 5, P = 1.000).

**S2 Fig. Ablation study results of adjuvant therapy candidacy prediction on the validation set.**

**Model evaluation on the test set**

In real-world applications, assessing models’ performance extends beyond the comparison provided by AUC. While AUC offers a comprehensive overview of a model's discrimination capability across various thresholds, specificity delineates the ability to identify true negatives and sensitivity measures the capacity to identify true positives. S2 Table shows these metrics and provides a deeper understanding of the features’ groups to predict adjuvant treatment.

**S2 Table. Sensitivity and specificity of logistic regression in the test set at Youden’s J-Score operating point selected on the validation set.**

|  | | Sensitivity | | Specificity | |
| --- | --- | --- | --- | --- | --- |
| All features | | 0.67 [0.65, 0.69] | | 0.59 [0.57, 0.61] | |
| Group 1: Demographics | | 0.66 [0.65, 0.67] | | 0.38 [0.37, 0.39] | |
| Group 2:  Social determinants of health | | 0.15 [0.14, 0.16] | | 0.80 [0.78, 0.81] | |
| Group 3: Comorbidities | | 0.45 [0.44, 0.46] | | 0.70 [0.69, 0.71] | |
| Group 4:  Clinical measurements | | 0.53 [0.51, 0.55] | | 0.58 [0.56, 0.60] | |
| Group 5:  Visual features | | 0.51 [0.50, 0.52] | | 0.89 [0.88, 89] | |

Note: Data in parenthesis represent 95% confidence intervals computed with 1,000 empirical bootstrap replicates of the models’ predictions.

## **S3 Appendix: Pretraining of BERT-based model for clinical records**

We employed the BERT architecture [4] for semi-supervised adjuvant therapy prediction learning, leveraging labeled and unlabeled data. In the first application of BERT to electronic health records (EHR) data, named BEHRT model [5], medical observations are considered as words, visits as sentences, and the records of each patient as a document. In our work, the data input to the BERT architecture was clinical records collected during individuals’ first visit to the assessment centers in the UK Biobank (UKB) dataset, without temporal information provided to the model. In other words, we did not insert a [SEP] token during pretraining, as all clinical data belongs to the same visit. We opted for this approach because longitudinal and sequential clinical records were available only for 45% of the UKB population (approximately 220,000 patients). Thus, we prioritized more patients to pretrain our model on, rather than limiting the pretraining data to over half a percent. The full pretraining cohort was randomly divided into bins of equal size, and subsequently, training, validation, and test sets were designed according to the specifications in S3 Table.

**S3 Table. Data split of the pretraining cohort.**

| UK Biobank full cohort (as of July 1, 2022) | 502,411 individuals |
| --- | --- |
| Training set (bins 0-60) | 301,279 individuals |
| Validation set (bins 61-80) | 100,938 individuals |
| Test set (bins 81-99) | 100,194 individuals |

The UKB clinical data was pre-processed according to the following steps:

- 1. Remove features with more than 90% missing values.
  2. Remove categorical features if they are too imbalanced (dominant category > 99%).
  3. Categorize continuous features into 5 bins using equally spaced quantiles.

Binning constants are calculated based on the training samples (bins 0-60) and are then applied to the entire preprocessing dataset. Binning plays a crucial role in reducing the number of tokens and consequently the vocabulary size of the language model. We used the FeatureX framework [6] to extract all features from the UKB. Given that many clinical features were self-reported and categorical, often with imbalanced proportions, certain information such as the family history of rare diseases or usage of vitamins and supplements was disregarded. In total, 1,143 clinical features were extracted, but 337 remained after the preprocessing steps described above.

Following the same common training procedure for transformer-based approaches, we first pretrained using Masked Language Modeling (MLM) to predict the original content of 12% of tokens that have been masked. This allows us to leverage larger datasets as this process does not need any supervision and train our model to better understand medical records' nuances. This framework, greatly popularized by the foundation (language) models [7], was directly adapted for the UKB data by masking the clinical tokens and evaluating the cross-entropy loss curve in the validation set. We performed a hyperparameter search on the token dimension, the learning rate, and the last linear layer dimension. We picked a combination of hyperparameters that led to the lowest cross-entropy loss (CELoss) on the validation set after 60 training epochs.

In addition to MLM, we performed a supervised training task to predict new diagnosis (DX) of 16 possible clinical conditions defined based on the Charlson comorbidity index [8]. To this end, we extracted all CCSR diagnosis codes from the General Practice tables from the UKB database and mapped them to one of the 16 conditions (S4 Table).

**S4 Table. Mapping of CCSR codes to 16 Charlson clinical conditions.**

| **Charlson clinical condition** | **CCSR code** |
| --- | --- |
| Myocardial infarction | CIR009_Acute myocardial infarction |
|  | CIR010_Complications of acute myocardial infarction |
| Congestive Heart Failure | CIR019_Heart failure |
| Peripheral Vascular Disease | CIR026_Peripheral and visceral vascular disease |
| Cerebrovascular Disease | CIR022_Sequela of hemorrhagic cerebrovascular disease |
|  | CIR021_Acute hemorrhagic cerebrovascular disease |
|  | CIR024_Other and ill-defined cerebrovascular disease |
|  | CIR025_Sequela of cerebral infarction and other cerebrovascular disease |
| Dementia | NVS011_Neurocognitive disorders |
| COPD | RSP008_Chronic obstructive pulmonary disease and bronchiectasis |
| Connective Tissue Disease | MUS025_Other specified connective tissue disease |
|  | MUS024_Systemic lupus erythematosus and connective tissue disorders |
| Peptic ulcer disease | DIG005_Gastroduodenal ulcer |
|  | DIG021_Gastrointestinal hemorrhage |
| Liver disease | DIG019_Other specified and unspecified liver disease |
| Diabetes Mellitus Chronic | \| END006_Diabetes mellitus, due to underlying condition, drug or chemical induced, or other specified type \| \| --- \| |
|  | END002_Diabetes mellitus without complication |
|  | END005_Diabetes mellitus, Type 2 |
|  | END003_Diabetes mellitus with complication |
| Hemiplegia paraplegia | NVS008_Paralysis (other than cerebral palsy) |
| Kidney disease | GEN003_Chronic kidney disease |
| Leukemia | NEO062_Leukemia - chronic myeloid leukemia (CML) |
|  | NEO061_Leukemia - chronic lymphocytic leukemia (CLL) |
|  | NEO064_Leukemia - all other types |
|  | NEO059_Leukemia - acute lymphoblastic leukemia (ALL) |
|  | NEO060_Leukemia - acute myeloid leukemia (AML) |
|  | NEO063_Leukemia - hairy cell |
| Lymphoma | NEO057_Hodgkin lymphoma |
|  | NEO058_Non-Hodgkin lymphoma |
| AIDS | INF006_HIV infection |
| Metastatic solid tumor | NEO017_Gastrointestinal cancers - liver |
|  | NEO021_Gastrointestinal cancers - all other types |
|  | NEO005_Head and neck cancers - nasopharyngeal |
|  | NEO072_Neoplasms of unspecified nature or uncertain behavior |
|  | NEO070_Secondary malignancies |
|  | NEO034_Female reproductive system cancers - fallopian tube |
|  | NEO056_Endocrine system cancers - all other types |
|  | NEO015_Gastrointestinal cancers - colorectal |
|  | NEO065_Multiple myeloma |
|  | NEO024_Sarcoma |
|  | NEO011_Cardiac cancers |
|  | NEO029_Breast cancer - ductal carcinoma in situ (DCIS) |
|  | NEO010_Head and neck cancers - all other types |
|  | NEO036_Female reproductive system cancers - vulva |
|  | NEO020_Gastrointestinal cancers - peritoneum |
|  | NEO053_Endocrine system cancers - adrenocortical |
|  | NEO006_Head and neck cancers - hypopharyngeal |
|  | NEO067_Mesothelioma |
|  | NEO049_Nervous system cancers - all other types |
|  | NEO016_Gastrointestinal cancers - anus |
|  | NEO031_Female reproductive system cancers - uterus |
|  | NEO022_Respiratory cancers |
|  | NEO050_Endocrine system cancers - thyroid |
|  | NEO013_Gastrointestinal cancers - stomach |
|  | NEO051_Endocrine system cancers - pancreas |
|  | NEO041_Male reproductive system cancers - penis |
|  | NEO038_Female reproductive system cancers - all other types |
|  | NEO030_Breast cancer - all other types |
|  | NEO001_Head and neck cancers - eye |
|  | NEO009_Head and neck cancers - tonsils |
|  | NEO004_Head and neck cancers - salivary gland |
|  | NEO035_Female reproductive system cancers - endometrium |
|  | NEO025_Skin cancers - melanoma |
|  | NEO071_Malignant neoplasm, unspecified |
|  | NEO007_Head and neck cancers - pharyngeal |
|  | NEO065_Multiple myeloma |
|  | NEO048_Nervous system cancers - brain |
|  | NEO018_Gastrointestinal cancers - bile duct |
|  | NEO028_Skin cancers - all other types |
|  | NEO008_Head and neck cancers - laryngeal |
|  | NEO055_Endocrine system cancers - pituitary gland |
|  | NEO032_Female reproductive system cancers - cervix |
|  | NEO039_Male reproductive system cancers - prostate |
|  | NEO037_Female reproductive system cancers - vagina |
|  | NEO046_Urinary system cancers - urethra |
|  | NEO012_Gastrointestinal cancers - esophagus |
|  | NEO047_Urinary system cancers - all other types |
|  | NEO040_Male reproductive system cancers - testis |
|  | NEO014_Gastrointestinal cancers - small intestine |
|  | NEO002_Head and neck cancers - lip and oral cavity |
|  | NEO003_Head and neck cancers - throat |
|  | NEO068_Myelodysplastic syndrome (MDS) |
|  | NEO069_Cancer of other sites |
|  | NEO023_Bone cancer |
|  | NEO054_Endocrine system cancers - parathyroid |

Individuals were followed up after index-date (day of the first visit to the assessment centers) for 730 days via record linkage to the (i) General Practice, (ii) the UK National Health Service Central Register, which provides information on cancer registrations and deaths, and (iii) inpatient data tables, which contains hospitalization information. We used data from all these sources to filter out patients that had any of the 16 diagnoses before the index-date The endpoint included in the analysis is the first diagnosis of a certain clinical condition within the follow-up period. The metric used to evaluate the task was the area under the receiver operating curve (AUC), and the macroAUC to aggregate the average of individual AUCs for each condition (S3 Fig). In addition, we used a binary cross-entropy with softmax loss (BCEwithLogitsLoss) to measure the reconstruction loss. Thus, the total pretraining loss was defined as a weighted sum of CELoss and BCEwithLogitsLoss:

Total loss = $\lambda_{1}$* ${Loss}_{MLM}+\lambda_{2}{Loss}_{DX}$

$${Loss}_{MLM}=CELoss= \left\{ l_{1}, \ldots, l_{N} \right\}^{T}, l_{n}=-\sum_{c=1}^{C} log\frac{\exp(x_{n,c})}{\sum_{i=1}^{C} exp(x_{n,i})}y_{n,c}$$

$${Loss}_{DX}= BCEwithLogitsLoss= \left\{ l_{1}, \ldots, l_{N} \right\}^{T}, l_{n}= \left[ y_{n}\cdot\log\sigma\left( x_{n} \right)+\left( 1-y_{n} \right)\cdot\log\left( 1-\sigma\left( x_{n} \right) \right) \right]$$

where $C$ is the number of classes, $x$ is the input, $y$ is the target, N is the batch size, $\lambda_{1}$ and $\lambda_{2}$ are scalar loss multipliers that are optimized during hyperparameter tuning.

**S3 Fig. Future diagnosis prediction performance in the validation set as a function of training epochs.**

Finally, when finetuning the pretrained model, we ensured feature compatibility by identifying overlapping features between the UKB and the KiTS datasets. Remarkably, all clinical features from the KiTS dataset could be linked to the same or similar features in the UKB dataset (S5 Table), except radiological size and the R.E.N.A.L. nephrometry score.

**S5 Table. Mapping of KiTS features to the UKB features.**

| **KiTS clinical feature** | **Matched UKB clinical feature** |
| --- | --- |
| Age at nephrectomy | Age at first visit |
| Gender | Gender |
| Body mass index | Body mass index |
| Smoking history: current smoker | Smoking status: current |
| Smoking history: never smoked | Smoking status: never |
| Smoking history: previous smoker | Smoking status: previous smoker |
| Age when quit smoking | Age of stopping smoking |
| Alcohol use: more than one daily | Alcohol intake frequency: daily or almost daily |
| Alcohol use: two or less daily | Alcohol intake frequency: daily or almost daily |
| Alcohol use: never or not in the last 3 months | Alcohol intake frequency: never |
| Alcohol use: quit in the last 3 months | Alcohol intake frequency: special occasions only |
| Cigarette pack years | Pack years of smoking |
| Chewing tobacco: never or not in the last 3 months | Current tobacco smoking: no |
| Chewing tobacco: currently chews | Current tobacco smoking: yes, on most or all days |
| Diabetes Mellitus with organ damage | Diabetes diagnosed by the doctor |
| Myocardial infarction | Self-reported heart attack: myocardial infarction |
| Congestive heart failure | Heart problems diagnosed by the doctor |
| Peripheral vascular disease | Self-reported peripheral vascular disease |
| Cerebrovascular disease | Cerebrovascular disease |
| Dementia | Cognitive impairment |
| COPD | Self-reported Chronic obstructive airways disease, COPD |
| Connective tissue disease | Self-reported connective tissue disorder |
| Peptic ulcer disease | Self-reported peptic ulcer |
| Uncomplicated diabetes mellitus | Self-reported diabetes |
| Chronic kidney disease | Self-reported renal: kidney problem |
| Hemiplegia from stroke | Serious medical condition: disability diagnosed by the doctor |
| Leukemia | Self-reported leukemia |
| Malignant lymphoma | Lymphoma |
| Localized solid tumor | Localized tumor |
| Metastatic solid tumor | Cancer diagnosed by the doctor |
| Mild liver disease | Self-reported alcoholic liver disease |
| Moderate to severe liver disease | Self-reported liver failure: cirrhosis |
| AIDS | Self-reported HIV |

## **S4 Appendix: Evaluation of top winners of KNIGHT Challenge**

The calibration curves of the four competitors’ models, the benchmark and average model are shown in S4 Fig.

**S4 Fig. Calibration curves on the test set.** The average model showed the smallest Brier score.

## **Supplemental References**

1. Heller N, Sathianathen N, Kalapara A, Walczak E, Moore K, Kaluzniak H, et al. The KiTS19 Challenge Data: 300 Kidney Tumor Cases with Clinical Context, CT Semantic Segmentations, and Surgical Outcomes [Internet]. arXiv; 2020 [cited 2024 Jun 2]. Available from: http://arxiv.org/abs/1904.00445

2. Carvalho JAM, Nunes P, Tavares-da-Silva E, Parada B, Jarimba R, Moreira P, et al. Impact of Positive Surgical Margins After Partial Nephrectomy. Eur Urol Open Sci. 2020 Oct 1;21:41–6.

3. Troyanskaya O, Cantor M, Sherlock G, Brown P, Hastie T, Tibshirani R, et al. Missing value estimation methods for DNA microarrays. Bioinformatics. 2001 Jun 1;17(6):520–5.

4. Devlin J, Chang MW, Lee K, Toutanova K. BERT: Pre-training of Deep Bidirectional Transformers for Language Understanding [Internet]. arXiv; 2019 [cited 2024 Jun 2]. Available from: http://arxiv.org/abs/1810.04805

5. Li Y, Rao S, Solares JRA, Hassaine A, Ramakrishnan R, Canoy D, et al. BEHRT: Transformer for Electronic Health Records. Sci Rep. 2020 Apr 28;10(1):7155.

6. Ozery-Flato M, Yanover C, Gottlieb A, Weissbrod, Omer, Parush Shear-Yashuv, Naama, Goldschmidt, Yaara. Fast and efficient feature engineering for multi-cohort analysis of EHR data. Inform Health Connect Citiz-Led Wellness Popul Health. 2017;181–5.

7. Bommasani R, Hudson DA, Adeli E, Altman R, Arora S, von Arx S, et al. On the Opportunities and Risks of Foundation Models [Internet]. arXiv; 2022 [cited 2024 Jun 5]. Available from: http://arxiv.org/abs/2108.07258

8. Charlson ME, Pompei P, Ales KL, MacKenzie CR. A new method of classifying prognostic comorbidity in longitudinal studies: Development and validation. J Chronic Dis. 1987 Jan 1;40(5):373–83.
