# Supplementary figures and images for: Preoperative kidney tumor risk estimation with AI: From logistic regression to transformer
Source: PLoS One. 2025 May 30;20(5):e0323240. doi: 10.1371/journal.pone.0323240 (PMC12124753; doi:10.1371/journal.pone.0323240)

Procedure Dates by Dataset

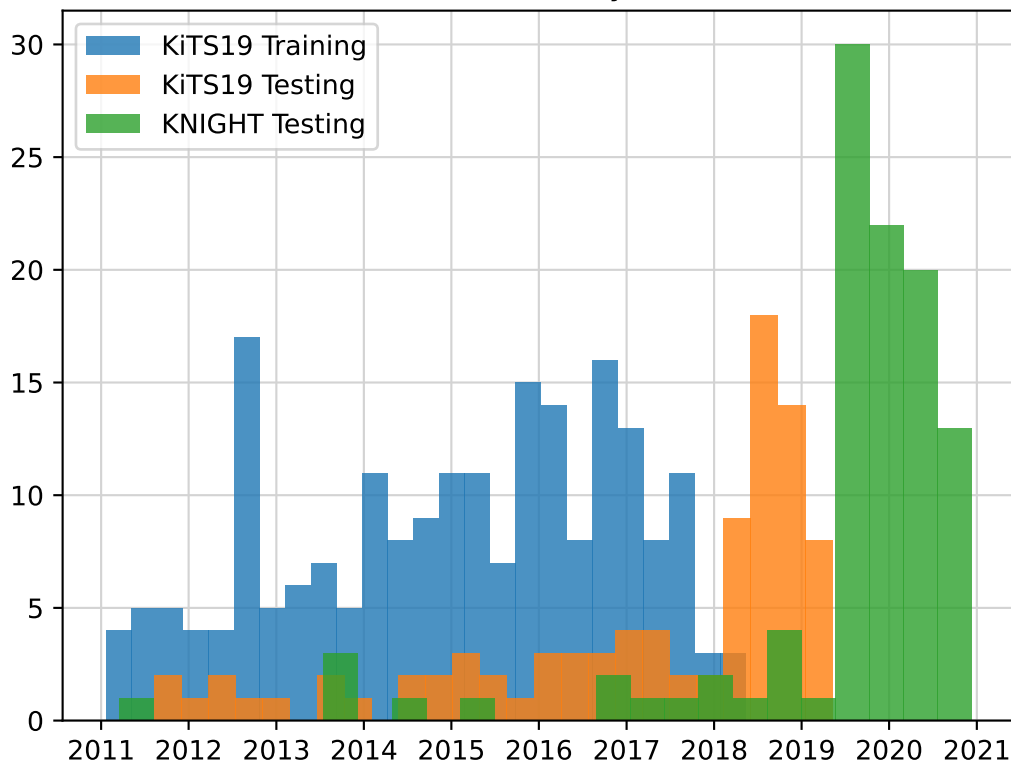

Supplement: S1 File — S1 Fig. The date span of the data used for the KNIGHT Challenge. S2 Fig. Ablation study results of adjuvant therapy candidacy prediction on the validation set. S3 Fig. Future diagnosis prediction performance in the validation set as a function of training epochs. S4 Fig. Calibration curves on the test set. The average model showed the smallest Brier score. S1 Table. Percentage of missing values in the dataset. S2 Table. Sensitivity and specificity of logistic regression in the test set at Youden’s J-Score operating point selected on the validation set. S3 Table. Data split of the pretraining cohort. S4 Table. Mapping of CCSR codes to 16 Charlson clinical conditions. S1 Appendix. The KiTS database. S2 Appendix. Clinical feature ablation study. S3 Appendix. Pretraining of BERT-based model for clinical records. S4 Appendix. Evaluation of top winners of KNIGHT Challenge. (ZIP) [file pone.0323240.s001.zip › S1_Fig.pdf]

Adjuvant therapy prediction on validation set

True Positive Rate

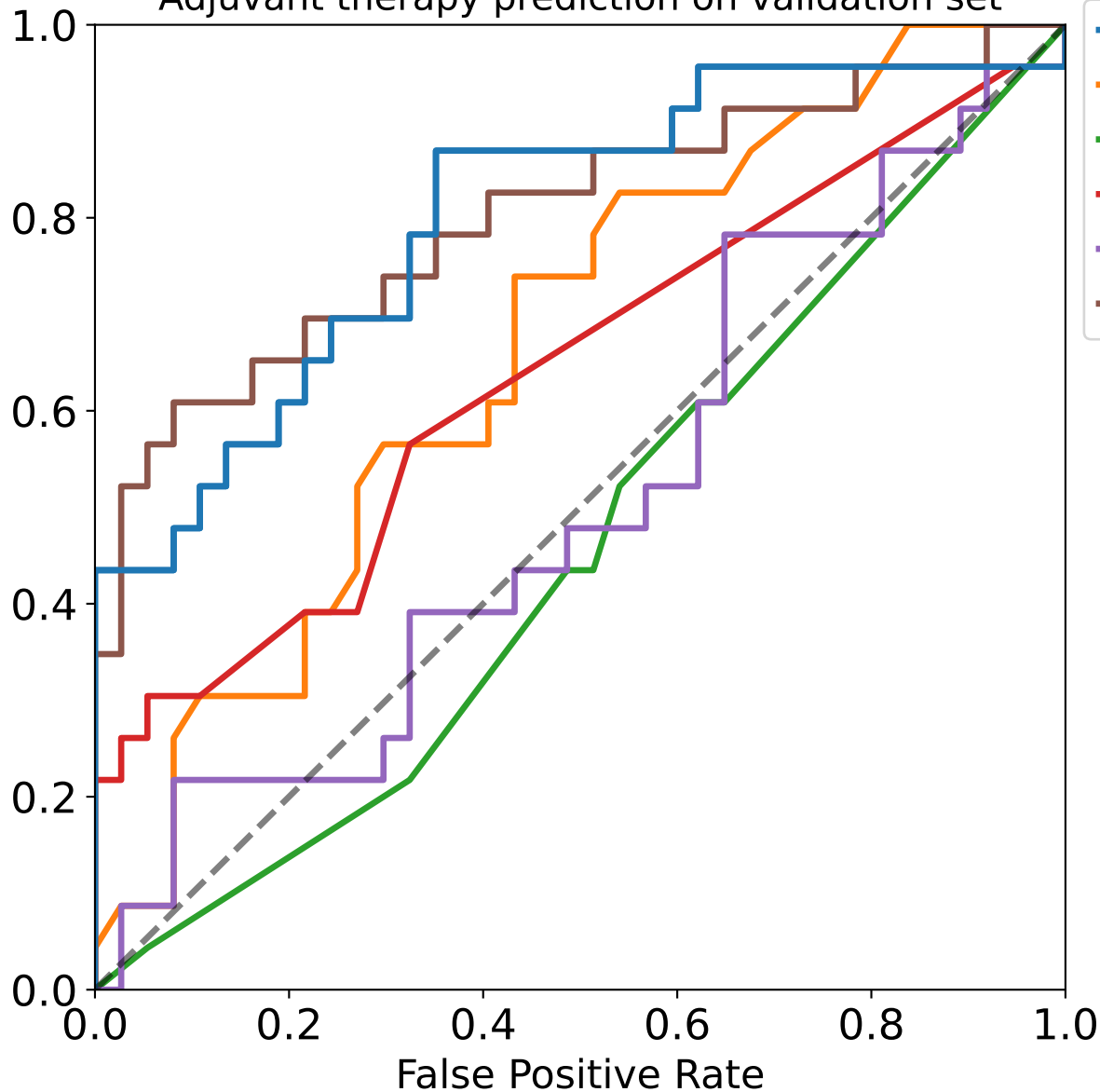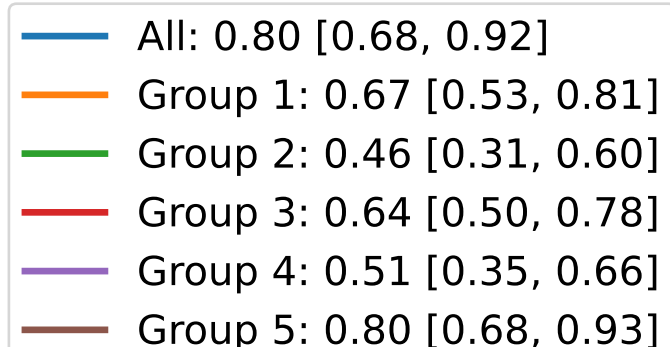

Supplement: S1 File — S1 Fig. The date span of the data used for the KNIGHT Challenge. S2 Fig. Ablation study results of adjuvant therapy candidacy prediction on the validation set. S3 Fig. Future diagnosis prediction performance in the validation set as a function of training epochs. S4 Fig. Calibration curves on the test set. The average model showed the smallest Brier score. S1 Table. Percentage of missing values in the dataset. S2 Table. Sensitivity and specificity of logistic regression in the test set at Youden’s J-Score operating point selected on the validation set. S3 Table. Data split of the pretraining cohort. S4 Table. Mapping of CCSR codes to 16 Charlson clinical conditions. S1 Appendix. The KiTS database. S2 Appendix. Clinical feature ablation study. S3 Appendix. Pretraining of BERT-based model for clinical records. S4 Appendix. Evaluation of top winners of KNIGHT Challenge. (ZIP) [file pone.0323240.s001.zip › S2_Fig.pdf]

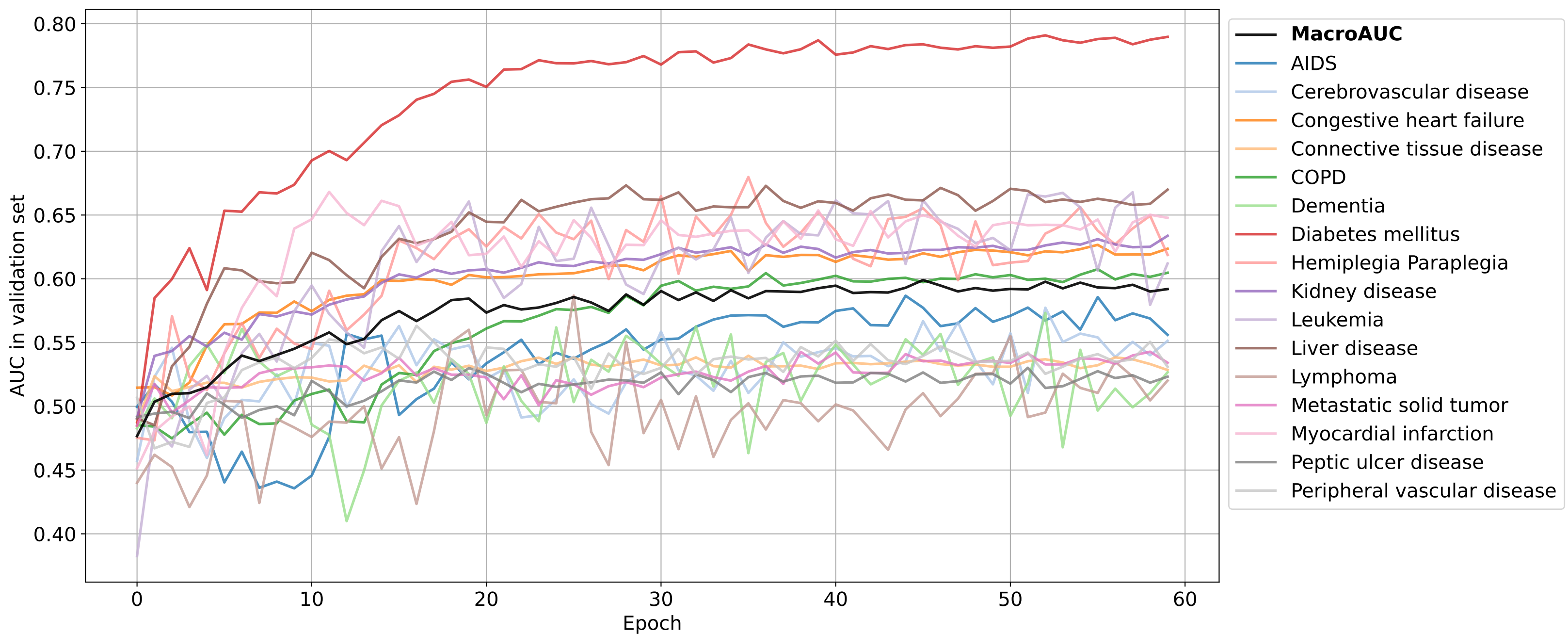

Supplement: S1 File — S1 Fig. The date span of the data used for the KNIGHT Challenge. S2 Fig. Ablation study results of adjuvant therapy candidacy prediction on the validation set. S3 Fig. Future diagnosis prediction performance in the validation set as a function of training epochs. S4 Fig. Calibration curves on the test set. The average model showed the smallest Brier score. S1 Table. Percentage of missing values in the dataset. S2 Table. Sensitivity and specificity of logistic regression in the test set at Youden’s J-Score operating point selected on the validation set. S3 Table. Data split of the pretraining cohort. S4 Table. Mapping of CCSR codes to 16 Charlson clinical conditions. S1 Appendix. The KiTS database. S2 Appendix. Clinical feature ablation study. S3 Appendix. Pretraining of BERT-based model for clinical records. S4 Appendix. Evaluation of top winners of KNIGHT Challenge. (ZIP) [file pone.0323240.s001.zip › S3_Fig.pdf]

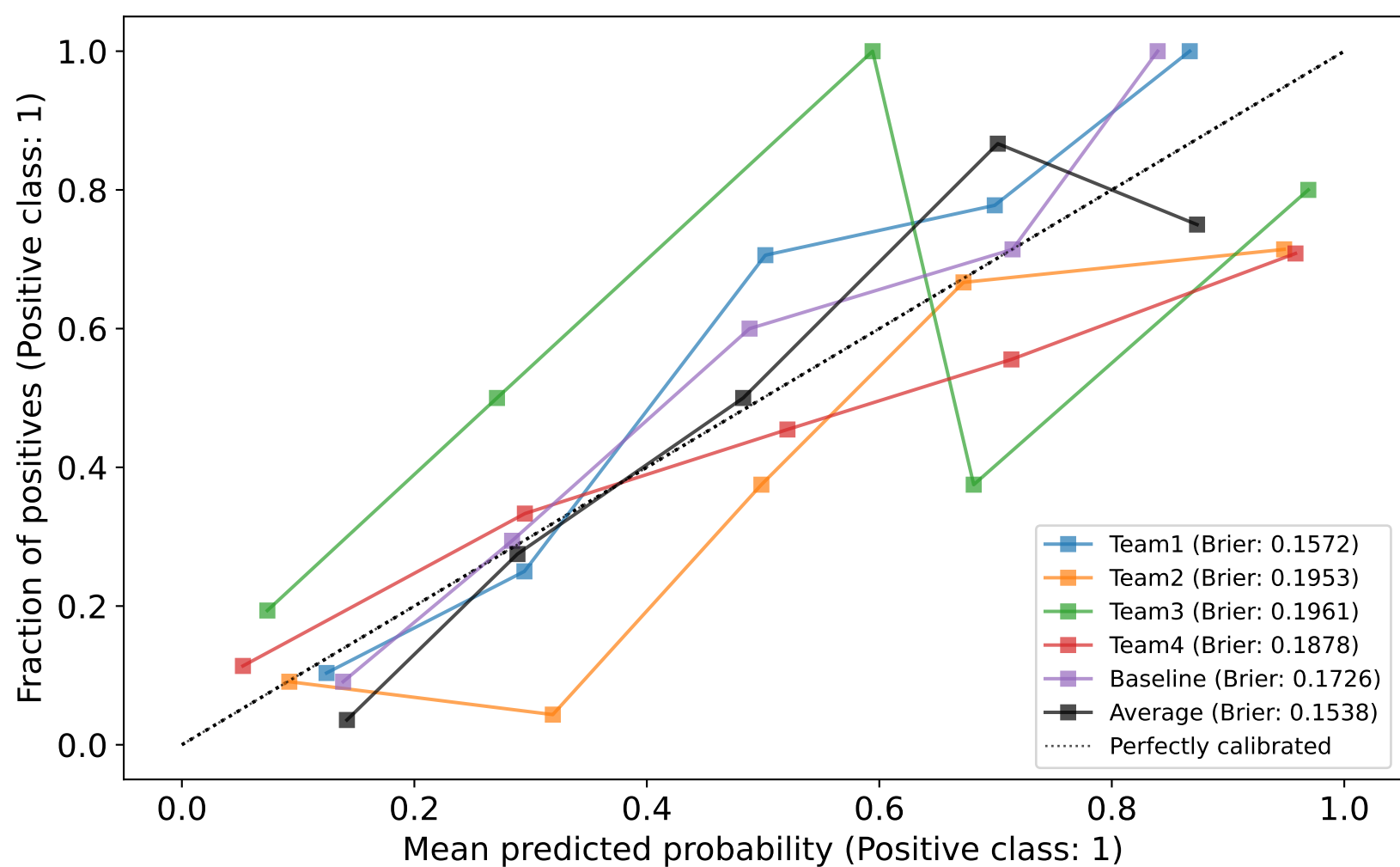

Supplement: S1 File — S1 Fig. The date span of the data used for the KNIGHT Challenge. S2 Fig. Ablation study results of adjuvant therapy candidacy prediction on the validation set. S3 Fig. Future diagnosis prediction performance in the validation set as a function of training epochs. S4 Fig. Calibration curves on the test set. The average model showed the smallest Brier score. S1 Table. Percentage of missing values in the dataset. S2 Table. Sensitivity and specificity of logistic regression in the test set at Youden’s J-Score operating point selected on the validation set. S3 Table. Data split of the pretraining cohort. S4 Table. Mapping of CCSR codes to 16 Charlson clinical conditions. S1 Appendix. The KiTS database. S2 Appendix. Clinical feature ablation study. S3 Appendix. Pretraining of BERT-based model for clinical records. S4 Appendix. Evaluation of top winners of KNIGHT Challenge. (ZIP) [file pone.0323240.s001.zip › S4_Fig.pdf]
